# A generalizable and easy-to-use COVID-19 stratification model for the next pandemic via immune-phenotyping and machine learning

**DOI:** 10.3389/fimmu.2024.1372539

**Published:** 2024-03-27

**Authors:** Xinlei He, Xiao Cui, Zhiling Zhao, Rui Wu, Qiang Zhang, Lei Xue, Hua Zhang, Qinggang Ge, Yuxin Leng

**Affiliations:** ^1^ Department of Intensive Care Unit, Peking University Third Hospital, Beijing, China; ^2^ Department of Pulmonary and Critical Care Medicine, Peking University Third Hospital, Beijing, China; ^3^ Department of Research Center of Clinical Epidemiology, Peking University Third Hospital, Beijing, China

**Keywords:** COVID-19, mass cytometry by time of flight (CyTOF), classical dendritic cells, lactate dehydrogenase, severity stratification, machine learning, decision-making

## Abstract

**Introduction:**

The coronavirus disease 2019 (COVID-19) pandemic has affected billions of people worldwide, and the lessons learned need to be concluded to get better prepared for the next pandemic. Early identification of high-risk patients is important for appropriate treatment and distribution of medical resources. A generalizable and easy-to-use COVID-19 severity stratification model is vital and may provide references for clinicians.

**Methods:**

Three COVID-19 cohorts (one discovery cohort and two validation cohorts) were included. Longitudinal peripheral blood mononuclear cells were collected from the discovery cohort (n = 39, mild = 15, critical = 24). The immune characteristics of COVID-19 and critical COVID-19 were analyzed by comparison with those of healthy volunteers (n = 16) and patients with mild COVID-19 using mass cytometry by time of flight (CyTOF). Subsequently, machine learning models were developed based on immune signatures and the most valuable laboratory parameters that performed well in distinguishing mild from critical cases. Finally, single-cell RNA sequencing data from a published study (n = 43) and electronic health records from a prospective cohort study (n = 840) were used to verify the role of crucial clinical laboratory and immune signature parameters in the stratification of COVID-19 severity.

**Results:**

Patients with COVID-19 were determined with disturbed glucose and tryptophan metabolism in two major innate immune clusters. Critical patients were further characterized by significant depletion of classical dendritic cells (cDCs), regulatory T cells (Tregs), and CD4**^+^** central memory T cells (Tcm), along with increased systemic interleukin-6 (IL-6), interleukin-12 (IL-12), and lactate dehydrogenase (LDH). The machine learning models based on the level of cDCs and LDH showed great potential for predicting critical cases. The model performances in severity stratification were validated in two cohorts (AUC = 0.77 and 0.88, respectively) infected with different strains in different periods. The reference limits of cDCs and LDH as biomarkers for predicting critical COVID-19 were 1.2% and 270.5 U/L, respectively.

**Conclusion:**

Overall, we developed and validated a generalizable and easy-to-use COVID-19 severity stratification model using machine learning algorithms. The level of cDCs and LDH will assist clinicians in making quick decisions during future pandemics.

## Graphical Abstract

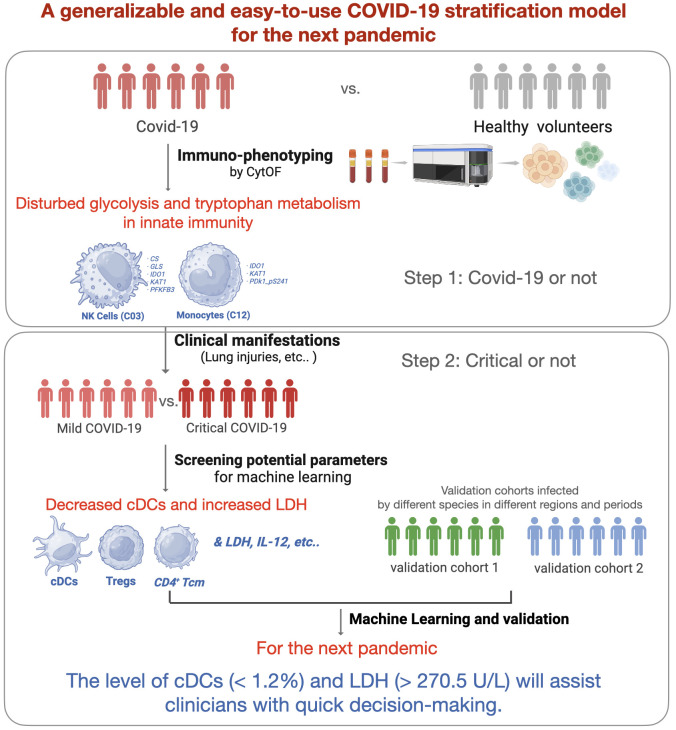

## 1 Introduction

The coronavirus disease 2019 (COVID-19) pandemic, caused by severe acute respiratory syndrome coronavirus 2 (SARS-CoV-2), has affected a global population exceeding 770 million individuals, leading to approximately 7.0 million fatalities (1). Although COVID-19 no longer constitutes a public health emergency of international concern, the whole world should review the lessons learned to prepare for the next pandemic (2). Better allocation of limited health resources, prediction of disease trajectories, and improvement of patient outcomes are essential during this pandemic. Therefore, the identification of critical patients is helpful for clinical management. Patients with critical COVID-19 have poor short- and long-term outcomes, including high in-hospital mortality and more post-acute COVID-19 syndromes (3). To improve preparedness and resilience to emerging threats, it is necessary to develop a generalizable COVID-19 severity stratification model, providing references for guiding the clinical management of the next pandemic.

Current COVID-19 stratification models are primarily based on a series of clinical manifestations, including vital signs, medical history, arterial blood gas results, laboratory tests, and chest imaging abnormalities (4, 5). In 2020, an easy-to-use COVID-19 severity score model was developed using eight commonly available parameters, which showed excellent performance in the identification of high-risk patients (6). However, the pathophysiology of these markers, which can foretell the prognosis of COVID-19 remains unclear. COVID-19 is characterized by a dysfunctional immune response against SARS-CoV-2 (7, 8). Immune-related biomarkers contribute to the understanding of disease progression and optimal treatments. Evidence suggests that severely ill patients show lymphocyte exhaustion (9–11), expansion of monocytes (12, 13), and cytokine storm (high levels of interleukin-6 [IL-6], C-reactive protein [CRP], and interferons) (14). By combining clinical manifestations and immunological biomarkers, a pathophysiology-based model will provide novel perspectives for clinical severity stratification.

Overall, we aimed to establish a generalizable COVID-19 severity stratification model using machine-learning methods. We aimed to elucidate the key immune signatures of patients with critical COVID-19 using mass cytometry by time of flight (CyTOF). By combining immune signatures and clinical parameters, the machine learning model is expected to improve our understanding of critical COVID-19 and provide references for quick decision-making during future pandemics.

## 2 Materials and methods

### 2.1 Study design

To prepare for the next COVID-19 pandemic, we established a clinical severity stratification model using machine learning with immune signatures. Three COVID-19 cohorts (one discovery cohort and two validation cohorts) and 16 age- and sex-matched healthy volunteers (negative for SARS-CoV-2 and virus-specific Immunoglobulin M [IgM] and Immunoglobulin G [IgG], as indicated by the reverse transcription-polymerase chain reaction [RT-PCR] test) were included in this study. According to the clinical severity classification criteria (**Supplementary Table S1**), which was modified from World Health Organization guidelines (2), patients in the discovery cohort were classified into mild and critical cases. We screened potential variables by longitudinally comparing the levels of anti-SARS-CoV-2 antibodies, inflammatory cytokines, plasma complement components, and cellular immune signatures between critical and mild cases. A self-designed 42-parameter panel, including nine energy metabolism enzymes, was applied to phenotypic immune signatures using CyTOF technology. The most clinically relevant immune signatures and plasma parameters were introduced into machine learning.

### 2.2 Patient cohorts

#### 2.2.1 Discovery cohort and sample collection

Patients who met the following inclusion criteria and were admitted to our surgical intensive care unit (ICU) between December 2021 and December 2022 were enrolled in the discovery cohort (n = 39, with 59 samples). Inclusion criteria were adults aged >18 years, first diagnosed with SARS-CoV-2 genome positivity using RT-PCR test in the previous 96 h, and sufficient remaining blood after regular laboratory tests on the first day post-admission. The exclusion criteria were as follows: age < 18 years; pregnancy; breastfeeding; existence of any pre-existing and transmissible diseases, such as human immunodeficiency virus, tuberculosis, and syphilis; mental illnesses; or taking psychotropic drugs. Basic information included comorbidities, in-hospital mortality, Murry lung injury score, and length of mechanical ventilation (**Table 1**).

**Table 1:** Clinical characteristics of COVID-19 discovery cohort.

Features	Mild (n = 15)	Critical (n = 24)	P-value
Age (yr.)	72.000 (63.00-76.00)	72.500 (69.00-77.75)	0.2020
Sex, male	12 (80.00%)	19 (79.17%)	>0.9999
Mortality	0% (0/15)	37.5% (9/24)	0.0069
Murry lung injury score	1.500 (0.00-4.00)	2.875 (2.37-3.29)	0.0110
Length of mechanical ventilation	0.000 (0.00-0.00)	1.000 (0.00-9.00)	0.0129
Length of ICU stay	6.000 (5.00-6.00)	10.000 (7.000-23.50)	0.0058
Comorbidities			
Circulatory diseases	11 (73.33%)	20 (83.33%)	0.6857
Endocrine diseases	7 (46.67%)	12 (50.00%)	>0.9999
Digestive diseases	5 (33.33%)	8 (33.33%)	>0.9999
Urological diseases	2 (13.33%)	5 (20.83%)	0.6857
Respiratory diseases	4 (26.67%)	5 (20.83%)	0.7110
Others	3 (20.00%)	5 (20.83%)	>0.9999

The statistical analyses were performed using Prism v.9.0 (GraphPad Software). For comparison between two groups, the gender, mortality, and comorbidities were evaluated using chi-square test and other clinical characteristics were evaluated by the Unpaired two-tailed Student’s t-test and data without a normal distribution were evaluated by the Mann-Whitey U-test. Data are presented as median with interquartile range.

Longitudinal (on days 1, 3, and 7 post-admission) blood samples were collected for analysis. Briefly, 2 mL peripheral blood samples were collected and delivered immediately to the lab at 4°C to gain the plasma and peripheral blood mononuclear cells (PBMCs). To avoid omitting potentially important information, both the absolute cell counts and relative cell proportion to PBMCs at all sampling points were analyzed in the present study.

#### 2.2.2 Validation cohort 1

To verify the key role of the most important immune subset (here, cDCs (C07)) in clinical severity stratification, we adopted public open data from Stephenson et al. (15). Briefly, single-cell data from mild (n = 26) and critical (n = 17) cases recruited from Addenbrooke’s Hospital, Royal Papworth Hospital, and University College London (UCL) Hospital were downloaded from https://covid19cellatlas.org/. The proportion of classical dendritic cells (cDCs) to PBMCs was filtered using the R package Seruat (4.0). According to the authors’ description, all patients were SARS-CoV-2 antigen-positive without active hematological malignancy or cancer, known immunodeficiency, sepsis from any cause, or blood transfusion within 4 weeks.

#### 2.2.3 Validation cohort 2

To verify the role of the most important systemic parameter (here, lactate dehydrogenase (LDH)) in clinical severity stratification, all the patients with complete clinical data admitted to other ICUs in our institution (Peking University Third Hospital) between December 2021 and December 2022 were retrospected (n = 840). Inclusion and exclusion criteria were the same with the discovery cohort.

### 2.3 Clinical laboratory data collection

Indices of interest, including the levels of inflammatory cytokines, complement components in plasma, and anti-SARS-CoV-2 antibodies, were extracted from electronic medical records (**Table 2**). Specifically, they were the systemic LDH, lactate, complement component 3 (C3), complement component 4 (C4), 50% hemolytic unit of complement (CH50), IgG, immunoglobulin A (IgA), IgM, immunoglobulin E (IgE), interleukin-1 (IL-1), interleukin-2 (IL-2), interleukin-4 (IL-4), interleukin-5 (IL-5), IL-6, interleukin-8 (IL-8), interleukin-9 (IL-9), interleukin-10 (IL-10), interleukin-12 (IL-12), interleukin-13 (IL-13), interleukin-17 (IL-17), interferon-α (IFN-α), interferon-γ (IFN-γ), tumor necrosis factor-α (TNF-α), granulocyte colony-stimulating factor, granulocyte macrophage colony-stimulating factor, vascular endothelial growth factor, macrophage inflammatory protein-1-α (MIP1-α), and monocyte chemotactic protein-1. All data were collected and verified by two experienced doctors.

**Table 2:** Laboratory characteristics of COVID-19 discovery cohort.

	Mild_Total (n = 24)	Critical_Total (n = 35)	P^#^	Mild_D1 (n = 15)	Critical_D1 (n = 15)	P^*^	Mild_D7 (n = 9)	Critical _D7 (n = 11)	P^$^
**LDH (U/L)**	239.40 ± 54.43	397.30 ± 219.20	0.0004	259.10 ± 46.84	487.40 ± 315.50	0.0032	190.00 ± 41.34	304.10 ± 104.70	0.0424
**Lactate (mmol/L)**	2.18 ± 0.39	2.79 ± 1.39	0.6961	2.18 ± 0.39	2.60 ± 1.56	0.8818	NA	2.63 ± 1.46	NA
**C3 (g/L)**	0.91 ± 0.19	0.88 ± 0.20	0.5294	0.94 ± 0.22	0.95 ± 0.15	0.8007	0.88 ± 0.16	0.78 ± 0.19	0.2319
**C4 (g/L)**	0.23 ± 0.11	0.22 ± 0.08	0.7375	0.24 ± 0.13	0.25 ± 0.08	0.2273	0.21 ± 0.06	0.17 ± 0.07	0.2627
**CH50 (U/ml)**	51.43 ± 12.89	45.37 ± 14.51	0.2027	50.57 ± 15.18	50.07 ± 11.55	0.3615	53.14 ± 6.99	39.30 ± 17.33	0.0999
**IgG (g/L)**	10.96 ± 2.65	12.51 ± 3.31	0.0820	11.31 ± 2.97	13.62 ± 3.97	0.1003	10.41 ± 2.10	10.71 ± 1.96	0.7556
**IgA (g/L)**	2.38 ± 1.03	2.72 ± 1.35	0.3269	2.65 ± 1.13	2.89 ± 1.41	0.6227	1.91 ± 0.66	2.17 ± 1.35	0.6322
**IgM (g/L)**	1.00 ± 0.53	0.99 ± 0.38	0.9375	1.00 ± 0.51	0.94 ± 0.38	0.7212	0.99 ± 0.59	1.01 ± 0.36	0.9549
**IgE (g/L)**	102.70 ± 145.50	168.60 ± 265.70	0.1419	116.40 ± 160.90	249.20 ± 366.40	0.2700	78.69 ± 120.00	85.16 ± 107.70	0.6334
**IL-1 (pg/ml)**	8.29 ± 9.31	8.30 ± 11.14	0.4043	7.66 ± 9.09	6.30 ± 7.58	0.4053	9.34 ± 10.12	13.52 ± 10.36	0.4470
**IL-2 (pg/ml)**	3.62 ± 1.75	3.18 ± 2.05	0.1525	3.53 ± 1.82	3.14 ± 1.75	0.5551	3.76 ± 1.71	3.84 ± 2.56	0.9409
**IL-4 (pg/ml)**	2.51 ± 1.05	2.65 ± 1.64	0.4385	2.66 ± 1.28	2.55 ± 1.66	0.3892	2.25 ± 0.45	3.11 ± 1.81	0.1848
**IL-5 (pg/ml)**	2.69 ± 1.52	2.71 ± 1.90	0.4104	2.06 ± 0.71	2.71 ± 1.92	0.8407	3.60 ± 1.93	3.03 ± 1.64	0.4938
**IL-6 (pg/ml)**	17.22 ± 13.49	116.50 ± 200.10	0.0257	18.00 ± 13.82	98.20 ± 109.90	0.2017	15.94 ± 13.63	70.36 ± 84.13	0.7103
**IL-8 (pg/ml)**	45.54 ± 67.81	115.10 ± 261.60	0.5127	45.66 ± 74.20	50.19 ± 119.40	0.6743	45.32 ± 59.86	248.10 ± 419.10	0.3702
**IL-9 (pg/ml)**	1.07 ± 0.37	1.211 ± 0.52	0.4920	1.19 ± 0.27	1.10 ± 0.40	0.6794	0.92 ± 0.46	2.34 ± 3.08	0.4762
**IL-10 (pg/ml)**	3.80 ± 1.80	3.58 ± 2.21	0.4242	3.86 ± 1.71	4.08 ± 2.85	0.7688	3.72 ± 2.04	2.69 ± 0.98	0.1703
**IL-12 (pg/ml)**	3.04 ± 1.93	2.20 ± 0.55	0.0189	3.11 ± 1.85	2.05 ± 0.63	0.0052	2.94 ± 2.16	2.25 ± 0.45	0.7197
**IL-13 (pg/ml)**	0.61 ± 0.48	0.68 ± 0.33	0.1265	0.72 ± 0.62	0.67 ± 0.19	0.1696	0.46 ± 0.09	0.59 ± 0.26	0.3879
**IL-17 (pg/ml)**	7.16 ± 5.06	11.25 ± 20.90	0.6749	7.33 ± 5.24	5.22 ± 3.19	0.1941	6.86 ± 5.05	8.79 ± 7.53	0.5336
**IFN-α (pg/ml)**	3.98 ± 2.45	3.34 ± 1.95	0.3066	3.88 ± 2.27	2.99 ± 1.48	0.2389	4.20 ± 2.84	4.33 ± 2.45	0.9193
**IFN-γ (pg/ml)**	5.70 ± 5.74	6.17 ± 5.77	0.9153	4.70 ± 3.02	6.42 ± 6.20	0.7340	7.33 ± 8.57	6.50 ± 5.24	0.7618
**TNF-α (pg/ml)**	3.97 ± 2.76	2.90 ± 2.11	0.1284	3.79 ± 2.67	3.18 ± 2.27	0.5299	4.28 ± 3.06	3.13 ± 2.24	0.3704
**GCSF (pg/ml)**	1.20 ± 0.33	5.12 ± 16.51	0.0963	1.32 ± 0.38	1.39 ± 0.39	0.7181	1.03 ± 0.12	1.68 ± 0.95	0.2825
**GM-CSF (pg/ml)**	7.94 ± 18.52	22.34 ± 41.72	0.1319	1.96 ± 0.10	3.03 ± 1.81	0.5478	2.24 ± 1.10	11.33 ± 26.64	0.4336
**VEGF (pg/ml)**	160.10 ± 62.70	285.60 ± 213.50	0.0813	181.10 ± 58.60	254.20 ± 151.30	0.2811	128.70 ± 62.22	284.70 ± 232.40	0.1483
**MIP1-α (pg/ml)**	28.17 ± 21.49	16.46 ± 13.31	0.1621	31.65 ± 26.92	13.18 ± 10.80	0.0705	22.93 ± 10.81	17.06 ± 15.16	0.2601
**MCP1 (pg/ml)**	123.60 ± 140.60	309.30 ± 806.10	0.8844	165.60 ± 172.80	198.20 ± 211.50	0.9813	60.55 ± 27.26	77.52 ± 71.16	0.9399

All statistical analyses were performed using Prism v.9.0 (GraphPad Software). For comparison between two groups, normally distributed data were evaluated by the unpaired two-tailed Student’s t-test and data without a normal distribution were evaluated by the Mann-Whitey U-test. Data were presented as mean ± SEM. P^#^, comparison between Mild_Total and Critical_Total; P^*^, comparison between Mild_D1 and Critical D1; P^$^, comparison between Mild_D7 and Critical_D7.

LDH, lactate dehydrogenase; C3, Complement component 3; C4, Complement component 4; CH50, 50% hemolytic unit of complement; IgG, Immunoglobulin G; IgA, Immunoglobulin A; IgM, Immunoglobulin M; IgE, Immunoglobulin E; IL-1, Interleukin-1; IL-2, Interleukin-2; Interleukin-4 (IL-4), IL-5, Interleukin-5; IL-6, Interleukin-6; IL-8, Interleukin-8; IL-9, Interleukin-9; IL-10, Interleukin-10; IL-12, Interleukin-12; IL-13, Interleukin-13; IL-17, Interleukin-17; INF-α, Interferon-α; IFN-γ, Interferon-γ; TNF-α, Tumor Necrosis Factor-α; GCSF, Granulocyte colony stimulating factor; GM-CSF, Granulocyte macrophage colony stimulating factor; VEGF, Vascular Endothelial Growth Factor; MIP1-α, Macrophage Inflammatory Protein-1-α; MCP1, Monocyte Chemotactic Protein-1.

### 2.4 Mass cytometry

PBMCs were isolated from peripheral blood using Ficoll density gradient centrifugation. To sort cell precipitates, they were combined with 5 mL of fluorescence-activated cell sorting (FACS) buffer (1×phosphate buffered saline supplemented with 0.5% bovine serum albumin) and centrifuged at 400×g for 5 min at 4°C. The supernatant was discarded and the cell precipitates were resuspended in FACS buffer. To examine the samples, the viability rate must be greater than 85%, and the number of cells must not be less than 3×10^6^.

To ensure homogeneous staining, approximately 2×10^6^–3×10^6^ PBMCs were used for each patient. PBMCs were stained with cisplatin (Fluidigm) (0.1 uL, 2 min, room temperature) for live/dead, washed with cell staining buffer (CSB) (Fluidigm), and spun down (300×g, 5 min, room temperature). PBMCs were then incubated with human TruStain FcX (BioLegend) for 10 min at room temperature. After incubation, PBMCs were stained with 50 uL surface receptor staining mix (30 min, room temperature) and washed twice with CSB (300×g, 5 min, room temperature). Next, the PBMCs were incubated with FixL buffer (Fluidigm) for 15 min at room temperature and washed twice with Perm-S buffer (Fluidigm) (800×g, 5 min, room temperature). PBMCs were stained with 50 uL intracellular mix (30 min, room temperature) and washed twice with CSB (800×g, 5 min, room temperature). PBMCs were fixed in 1 mL 1.6% paraformaldehyde. Samples were fixed and permeabilized by incubating 1 mL Fix and Perm buffer (Fluidigm) with 1 uL nucleic acid Ir-Intercalator (Fluidigm) overnight at 4°C. Metal-conjugated antibodies and other reagents are listed in **Supplementary Table S2**.

### 2.5 CyTOF data acquisition

Before acquisition, PBMCs were washed twice with CSB and resuspended at a concentration of 1.1×10^6^ cells/mL in the Cell Acquisition Solution (Fluidigm) containing 10% EQ Four Element Calibration Beads (Fluidigm). PBMCs were acquired using a Helios CyTOF Mass Cytometer (Fluidigm) equipped with a SuperSampler fluidics system (Victorian Airships), and data were collected as previously described. fcs files.

### 2.6 CyTOF data analysis

After acquisition, data were concatenated using the fcs concatenation tool from Cytobank and manually gated to retain live, singlet, and valid immune cells. CytoNorm was used in two steps according to the instructions provided in the R library CytoNorm to normalize the data (16). For the downstream analysis, the fcs files were loaded into R. The signal intensities for each channel were arcsinh-transformed with a cofactor of 5 (x_transf = asinh(x/5)). To visualize high-dimensional data, t-distributed stochastic neighbor embedding analysis (t-SNE) (17) and flow self-organizing map (FlowSOM) (18) algorithms were performed on all samples. Approximately 10,000 cell events in each sample were pooled and included in the t-SNE analysis, with a perplexity of 30 and theta of 0.5. The R t-SNE package for the Barnes Hut implementation of the t-SNE was used in this study. To study the developmental trajectory of natural killer (NK) cells and classical monocytes, dynamic immunometabolic states and cell transitions were analyzed using the Monocle algorithm (19). Data are displayed using the ggplot2 R package.

### 2.7 Machine learning strategies

Since the target variable (clinical severity) for model training was labelled data, provided by clinical experts. The supervised learning methods are more appropriate than unsupervised-, semi-supervised-, and reinforcement learning methods. By comparing the advantages of different supervised methods (20–30)(**Supplementary Table S3**), we finally employed AdaBoost, Back Propagation, Gradient Boosting Decision Tree, Random Forest, and Support Vector Machine algorithms to construct classifiers for discriminating patients with critical COVID-19 from mild ones. The important immune and systemic features (cDCs and LDH) were introduced to the model as inputs. Five-fold cross-validation (with four folds for training and one-fold for validation) and external validation were performed. For five-fold cross-validation, all the training data were randomly split into five parts. Each part was considered as the training part and the others were used for validation. Here, we performed the five-fold cross-validation five times and the averaged values of AUC were adopted. For the external validation, Back Propagation algorithm was performed.

### 2.8 Statistical analysis

Statistical analyses were performed using the R software (v.4.0.4). The normality of patient data was tested using the Shapiro–Wilk normality test. Statistically significant differences between phenotypes were calculated using two-sided multiple Student’s t-tests for variables with a normal distribution and Wilcoxon rank-sum tests for other variables. Spearman’s correlation analysis was performed on significantly different clusters, cytokines, and clinical indicators to assess their correlations using the R package stats (4.1.0). Receiver operating characteristic (ROC) analysis was performed with the R package pROC (1.16.2), and a heatmap was generated with the R package ggplot2 (4.0.5).

## 3 Results

### 3.1 Basic information and systemic inflammatory responses of the discovery cohort

A total of 39 individuals diagnosed with COVID-19 (15 mild and 24 critical cases) admitted to our ICU were included in cohort 1 as the discovery cohort to determine potential predictive parameters. As shown in **Table 1**, the basic information of the critical and mild cases was comparable. The Murry lung injury score, length of mechanical ventilation, and length of ICU stay were significantly high in critical cases (**Table 1**). Longitudinal comparisons of inflammatory cytokines, antibodies, and complement components revealed that systemic IL-6, IL-12, and LDH levels were important in distinguishing mild cases from critical cases. The variation trends in these parameters were consistent across all sampling points (**Table 2**).

### 3.2 Cellular immunometabolic characteristic of patients with COVID-19 differed from healthy volunteers

**Figure 1:**
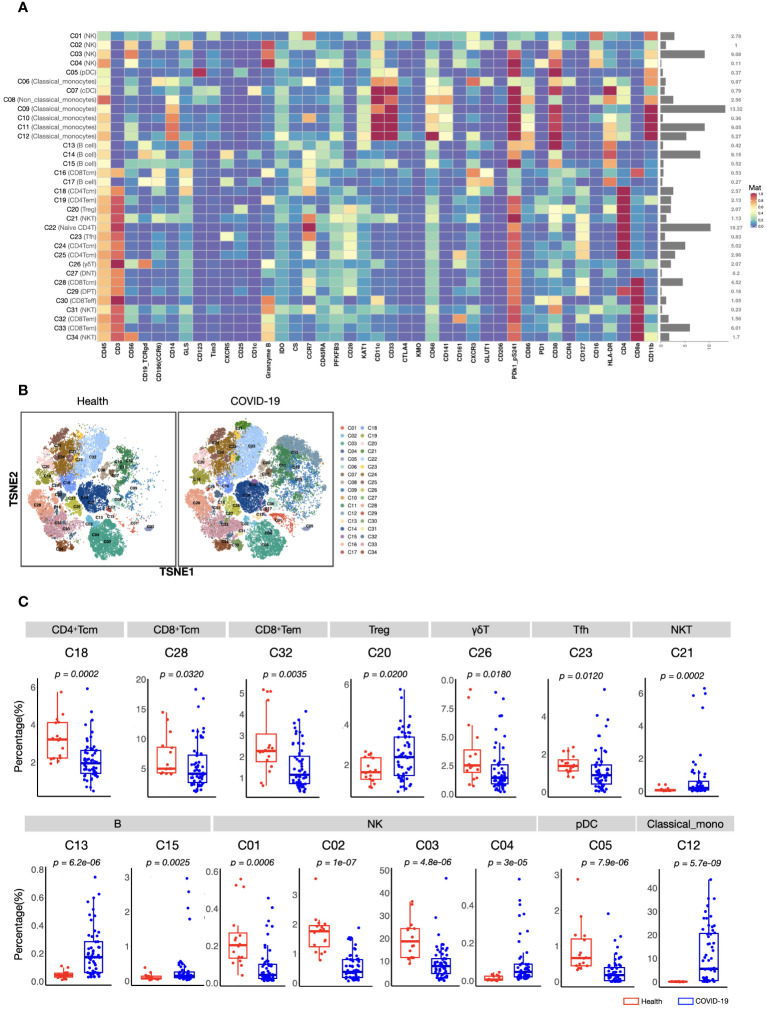
CyTOF analysis of peripheral immune cell subsets in patients with COVID-19 and healthy volunteers. **(A)** Heatmap showing normalized expression of 42 markers for 34 identified clusters. Relative frequency of each cluster is displayed as the right bar. **(B)** T-SNE maps displaying the relative distribution of 34 identified clusters across the groups. Immune cells were pooled from 30,000 cellular events in each sample. **(C)** Boxplots showing the frequencies of differed cell clusters between patients with COVID-19 and healthy volunteers. The center, box and whiskers of the boxplot represent the median, IQR and 1.5 × IQR, respectively. The t-test was used for normally distributed data and the Mann–Whitney U-test was used for non-normally distributed data.

To acquire a full landscape of the immune signatures of PBMCs and identify the potentially important clusters for the stratification of COVID-19, we performed CyTOF analysis with a 42-parameter panel (consisting of 33 surface markers and 9 intracellular metabolic markers) (**Supplementary information, Figure S1**). The obtained data were subjected to a FlowSOM clustering algorithm and t-distributed stochastic neighbour embedding (t-SNE) analysis, which enabled the identification of distinct clusters representing different immune cell types. According to the dimensional reduction results of the marker expression level, 34 clusters were obtained (**Figure 1A**). Then, to provide reference for other similar studies, which may apply different panels, we further classified these 34 clusters into “eleven major immune cell populations” (CD4^+^ T, CD8^+^ T, γδT, DPT, DNT, pDC, cDC, NK, NKT, B, and Monocytes), which were often studied (**Supplementary information, Table S4; Figure S2**).

**Figure 2:**
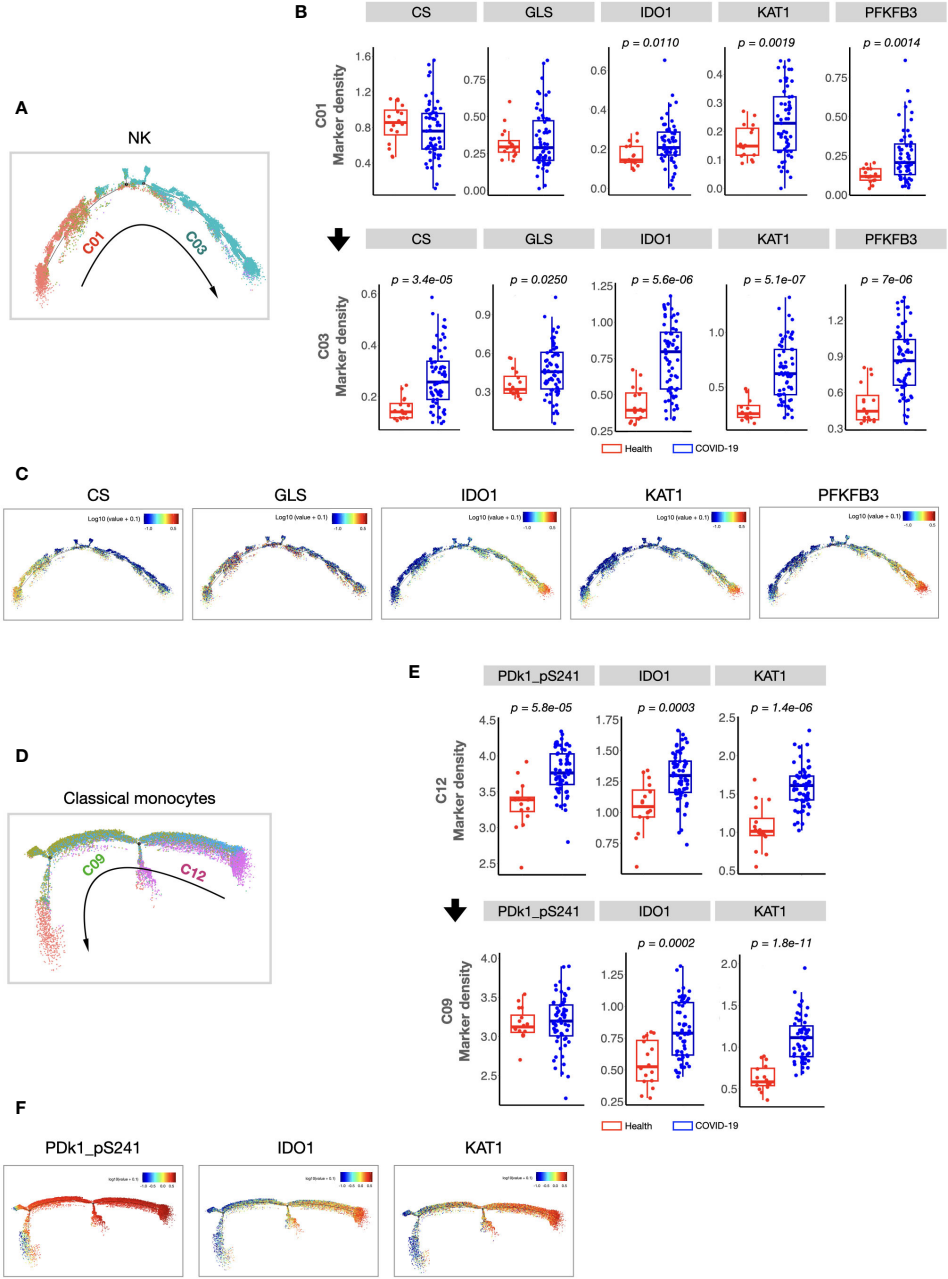
Cellular immunometabolic characteristics of COVID-19-specific immune subsets. **(A)** Monocle 2 trajectory analysis of NK cells. The monocle plot displays NK cells color-coded by different NK cell clusters. The arrow indicates the pseudotime trajectory of NK cells from a healthy state to COVID-19 infection. C01 was localized at the beginning of the pseudotime trajectory, whereas C03 was at the end of the trajectory. **(B)** Boxplots showing the density of the cellular metabolic markers (CS, GLS, IDO, KAT1, and PFKFB3) of C01 and C03. **(C)** Monocle 2 trajectory analysis of cellular metabolic markers of NK cells. Each dot represents one cell and colors represent the expression levels of indicated markers. **(D)** Monocle 2 trajectory analysis of classical monocytes. The monocle plot displays classical monocytes color-coded by different classical monocytes clusters. The arrow indicates the pseudotime trajectory of classical monocytes from healthy state to COVID-19 infection. **(E)** Boxplots showing the density of the cellular metabolic markers (CS, GLS, IDO, KAT1, and PFKFB3) of the C12 and C09. **(F)** Monocle 2 trajectory analysis of cellular metabolic markers of classical monocytes. Each dot represents one cell, and colors represent the expression levels of indicated markers. The center, box and whiskers of the boxplot represent the median, IQR and 1.5 × IQR, respectively. The t-test was used for normally distributed data and the Mann–Whitney U-test was used for non-normally distributed data.

We found that the composition of PBMCs in patients with COVID-19 varied significantly from that in healthy volunteers. The total counts of PBMCs (in per millilitre of peripheral blood) and the counts of the main immune cell types, such as T, B, and NK cells, of patients with COVID-19 decreased significantly. However, the number of monocytes increased (**Supplementary information, Figure S2**). Comparison of the percentages of all defined 34 clusters further confirmed that, fifteen immune cell subsets were significantly differed between COVID-19 patients and healthy volunteers (**Figures 1B, C**). Most of these subsets were acquired immune cell subsets and were significantly decreased in COVID-19. In addition, variations in two major innate immune cell subsets (NK cells (C03) and classical monocytes (C12), with the average percentages more than 5% in healthy volunteers) were also found (**Figures 1B, C**). As the host innate immunity is the first line of defense, we further investigated these two subsets’ metabolic status. As shown in **Figure 2**, the metabolic markers participating in the process of glucose (such as CS, GLS, PFKFB3, and PDk1_pS241) and tryptophan metabolism (IDO1 and KAT1) were significantly altered in both NK cells (C03) and classical monocytes (C12). The developmental trajectories further demonstrated that under COVID-19, NK cells gradually transformed from C01 to C03, namely, from a relative metabolic steady state to a disturbed state with decreased oxidative phosphorylation but boosted glycolysis and tryptophan catabolism (**Figures 2A–C**). For classical monocytes, C12 gradually transformed to C09, namely, to tryptophan exhaustion (**Figures 2D–F**).

### 3.3 Distinct cellular immune signatures of critical COVID-19 were identified compared with mild cases

**Figure 3:**
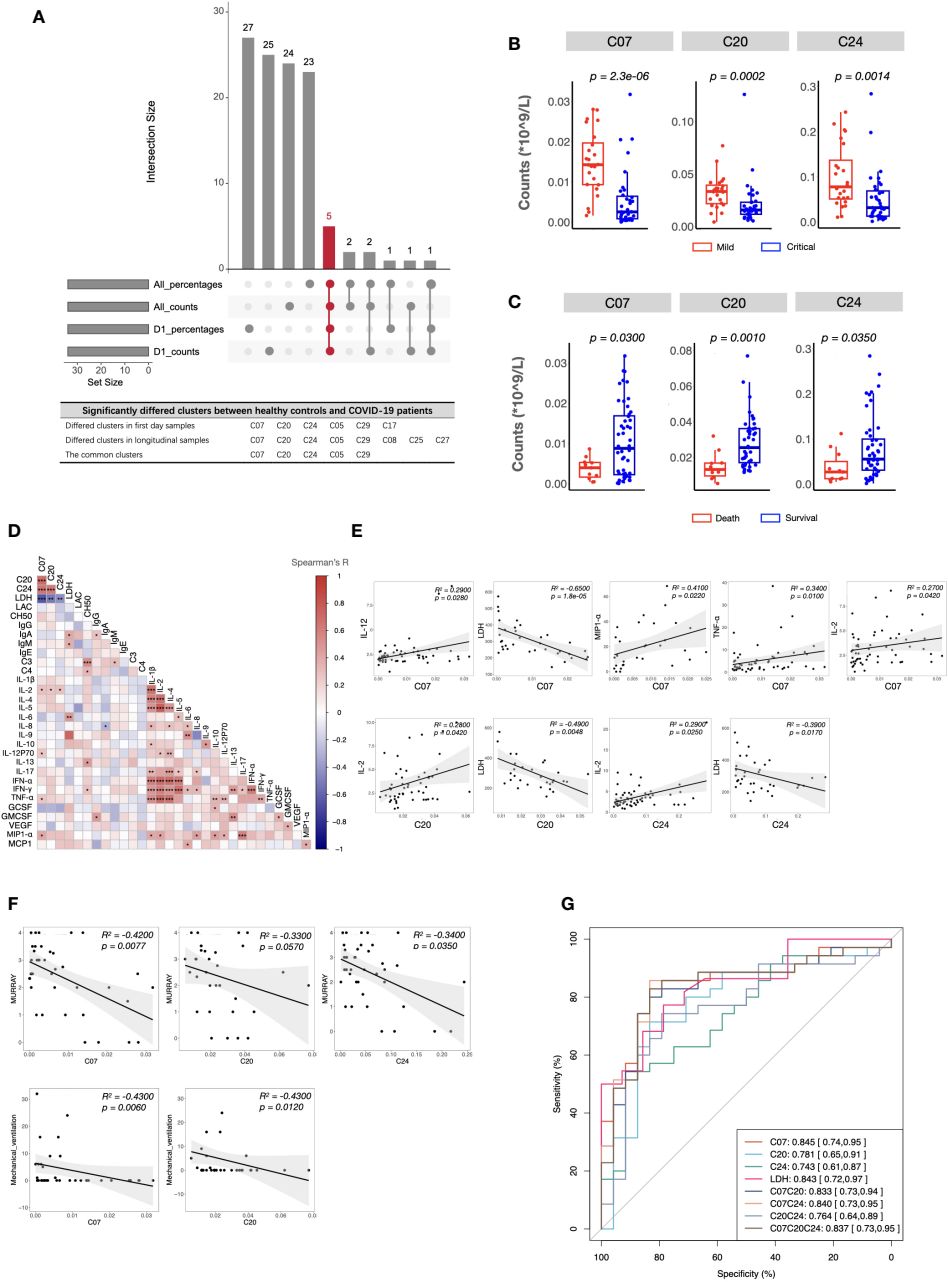
Immune and clinical characteristics of patients with critical COVID-19. **(A)** The candidate clusters distinguishing patients with critical COVID-19 from mild ones. **(B, C)** Boxplots depicting the cell counts of significantly differed clusters between patients with mild and critical COVID-19 **(B)**, and between survived and dead patients **(C)**. **(D)** Heatmap showing Spearman’s correlations between the counts of critical COVID-19 key immune clusters and clinical laboratory parameters in all samples. Colors represent Spearman’s correlation coefficient. **(E, F)** Scatterplots showing correlations between the counts of critical COVID-19 key immune clusters and critical clinical laboratory parameters **(E)**, Murray scores, and length of mechanical ventilation days **(F)**. **(G)** ROC analysis predicting COVID-19 severity using the counts of critical COVID-19-specific clusters and the level of LDH. The center, box and whiskers of the boxplot represent the median, IQR and 1.5 × IQR, respectively. The t-test was used for normally distributed data and the Mann–Whitney U-test was used for non-normally distributed data.

As described in the Methods section, to identify the important clusters distinguishing critical cases from mild cases, we compared the cell counts and percentages of each cluster within PBMCs at all sampling points. In total, five candidate clusters were found, and the differences in cDCs (C07), Tregs (C20), CD4^+^ Tcm (C24), pDCs (C05), and DPT (C29) were shared by the results from all sampling points and the first day samples (**Figure 3A**). As the percentages of pDCs and DPT were below 0.5%, they were not considered in subsequent analyses. Next, we investigated whether these clusters were associated with clinical parameters and prognosis. The results demonstrated that the counts of cDCs, Tregs, and CD4^+^ Tcm were significantly decreased in the critical cases and patients who ultimately died (**Figures 3B, C**). Their levels were positively or negatively correlated with systemic parameters, lung injuries, and the length of mechanical ventilation (**Figures 3D–F**, and **Supplementary information, Table S5**). Within each severity group, the longitudinal analysis showed that the counts of these three clusters were not significantly different among different sampling points (**Supplementary information, Figure S3**). These findings indicated that altered cDCs, Tregs, and CD4^+^ Tcm were stable/sensitive predictive biomarkers because their level wouldn’t be significantly influenced by sampling timing and/or transient condition relief. Specifically, cDCs was the most important cluster, negatively correlated with LDH and positively correlated with IL-2, IL-12, TNF-α, and MIP1-α (**Figure 3E**). Receiver operating characteristic analysis further revealed that the single variable cDCs was effective in predicting critical COVID-19 (**Figure 3G**). And the level of LDH was the most important systemic parameter because of its strong negative correlation with cDCs, Tregs, and CD4^+^ Tcm (**Figure 3E**).

### 3.4 Development and validation of clinical severity stratification models based on the immune signatures and plasma parameters of patients with critical COVID-19

**Figure 4:**
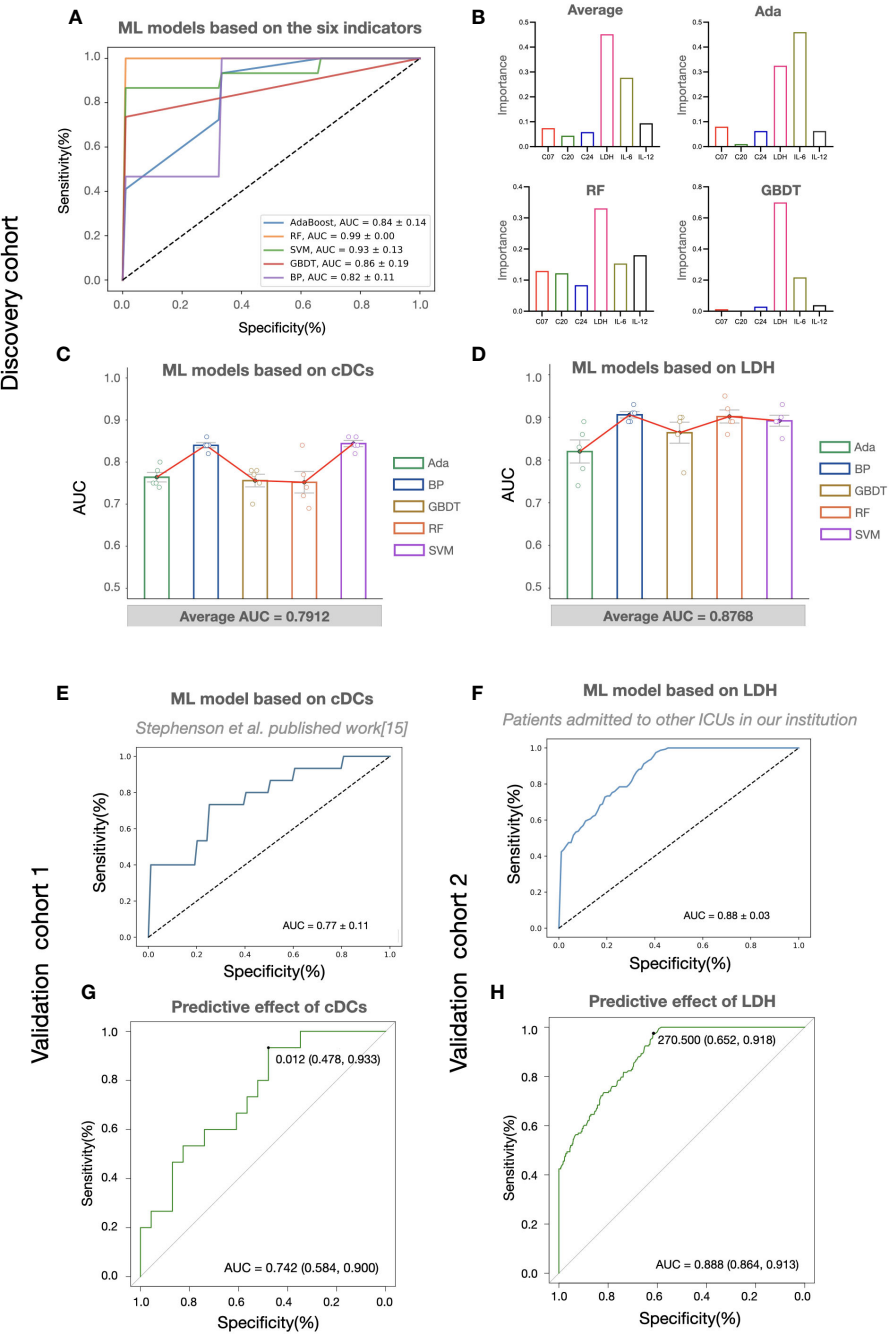
The predictive effects of cDCs and LDH on COVID-19 severity stratification. **(A)** Performances of COVID-19 severity stratification models based on the six candidate indicators (C07, C20, C24, LDH, IL-6, and IL-12) using five different machine learning algorithms in the discovery cohort. **(B)** The bar charts showing the contributions of six indicators in Ada, RF, and GBDT, as well as the averaged contributions of the six indicators across the three models. **(C, D)** Performances of COVID-19 severity stratification models based on the counts of cDCs (C07) **(C)** and the level of LDH **(D)** using five different machine learning algorithms in the discovery cohort. Each dot represents an AUC value of 5-fold cross-validation, and the bar shows the averaged AUC values from 5 runs. **(E, F)** Performances of COVID-19 severity stratification models based on the cDCs (C07) in validation cohort 1 **(E)**, and LDH in validation cohort 2 **(F)** by Back Propagation algorithm. **(G, H)** ROC analysis of cDCs **(G)** and LDH **(H)** for the COVID-19 stratification in the validation cohorts.

Considering the potential of machine learning for disease severity stratification, we developed clinical severity stratification models based on important key clusters (cDCs, Tregs, and CD4^+^ Tcm) and systemic parameters (LDH, IL-6, IL-12). As we expected, machine learning models with six parameters as inputs showed good effects in predicting clinical severity (**Figure 4A**). Among these parameters, cDCs and LDH were the most important immune signature and systemic signature, respectively (**Figure 4B**). The model using cDCs and LDH as individual input also performed well, with an average AUC of approximately 0.8 in the discovery cohort (**Figures 4C, D**). The validation of machine learning models with single input (with Back Propagation algorithm) further demonstrated that the clinical severity stratification model based on single cDCs had an AUC of 0.77 (**Figure 4E**). And the model based on systemic LDH had an AUC of 0.88 (**Figure 4F**). Notably, patients in validation cohort 1 were recruited in 2020 and infected with a different strain compared with the patients in the discovery cohort. These results indicate that our models, based on single biomarker (cDCs or LDH), performed well in COVID-19 severity stratification, with good robustness and generalization.

### 3.5 Reference limits of cDCs and LDH as biomarkers for predicting critical COVID-19

To provide detail reference for clinicians in quick decision-making for the next pandemic, we analyzed the effect of cDCs and LDH in severity prediction in validation cohorts and tried to find out the optimal reference limits. In validation cohort 1 (adopted from *Stephenson et al.’s published work* (15)), the proportion of cDCs decreased in critically ill participants across the three UK centers (**Supplementary information, Figures S4A–C**). The percentage of cDCs showed good effects in predicting clinical severity (AUC = 0.74, **Figure 4G**). The optimal cutoff point was 1.2%, and the sensitivity was 0.93 (95% CI 0.70-0.99). In the validation cohort 2 (adopted from Peking University Third Hospital), similar with the findings in the discovery cohort, significant increase of LDH (**Supplementary information, Figure S4D**) and its predictive effect was found (AUC = 0.89, **Figure 4H**). The cutoff point was 270.5 U/L and the sensitivity was 0.92 (95% CI 0.86-0.95). Accordingly, the reference limits of cDCs and LDH for critical COVID-19 were less than 1.2% and more than 270.5 U/L.

## 4 Discussion

Since the beginning of the SARS-CoV-2 pandemic, numerous researchers have provided important perspectives on the underlying mechanisms of COVID-19 and have developed severity stratification models (31). To provide novel insights and better preparations for the next pandemic, we developed a severity stratification model with good generalizability based on the pathophysiology of COVID-19. Through integrative analysis of immune signatures and clinical manifestations in critical participants, we found that cDCs and systemic LDH levels were the most important factors that determined severity stratification (Figure3G). The key roles of the two indicators were validated using two cohorts. Notably, the machine learning models based on the level of cDCs and LDH showed great potential for predicting critical cases in cohorts infected with different strains (**Figures 4E, F**). The reference limits of cDCs and LDH as biomarkers for predicting critical COVID-19 were 1.2% and 270.5 U/L, respectively (**Figures 4G, H**).

According to the current World Health Organization criteria, critical and severe COVID-19 are identified by a bundle of clinical features, including chest imaging characteristics, arterial blood gas parameters, and other clinical symptoms and signs (2). A progressive decrease in peripheral blood lymphocytes, an increase in IL-6, CRP, procalcitonin, and D-dimer are considered biomarkers for COVID-19 severity based on guidelines (32). In the present study, we detected that LDH showed great potential in the early identification of patients with critical COVID-19 (33–36). Although LDH is considered a nonspecific biomarker of inflammation, its elevation is associated with poor outcomes, possibly reflecting the severity of lung damage (37, 38). Furthermore, a large meta-analysis suggested that increased LDH levels following infection correlated with the post-acute respiratory sequelae of COVID-19, showing great potential in predicting long-term COVID-19 (39).

Certain profound immunity alterations took place during COVID-19 infection, and the depletion and dysfunction of lymphocytes were described as the most classical signatures of critical COVID-19 in most articles. Although we also observed decreased Tregs and CD4^+^ central memory T cells in critical cases, the counts of cDCs contributed the most to predict clinical severity. Several studies have demonstrated the reduction and dysfunction of cDCs in critical COVID-19 (40, 41), our study was supported by these results and further emphasized its key role in severity stratification models. As highly efficient antigen-presenting cells, DC are the key link between innate and adaptive immunity. Several ongoing clinical trials have been assessing the safety and efficiency of DC-based vaccines against SARS-CoV-2 (42, 43). DCs can activate T cell responses and save adjacent cells by secreting type I interferons (44). However, some limitations of DC-based vaccines, such as toxicity, allergenicity, and the possibility of DCs phenotype alterations, have not been resolved (42). Therefore, further studies on DCs as treatable traits are required.

Researches have demonstrated that comorbidities have an impact on the severity of COVID-19 in patients (45). SARS-CoV-2 is more likely to affect older men with comorbidities (46), and the presence of comorbidity is more common in patients with severe COVID-19 (45) than mild patients. Patients with diabetes, cardiovascular diseases, and respiratory diseases, are more likely to present more severe symptoms and complications (33, 47). However, our patients with COVID-19 were all from specialty ICU, who tended to be with a poor underlying functional status and with more comorbidities (**Table 1**). Accordingly, our conclusions may not be as applicable to those without comorbidities or with a healthy status. This is a limitation of our study, and future studies are encouraged to address this issue.

In summary, we established a severity stratification model for COVID-19 based on integrative analysis of immune signatures and clinical laboratory parameters. This machine-learning model was validated in two cohorts infected with different strains, demonstrating its generalizability and robustness. We hope that our analysis will be beneficial for the early identification of high-risk patients with COVID-19 and provide some references for the next pandemic.

## Data availability statement

The original contributions presented in the study are included in the article/**Supplementary Material**. Further inquiries can be directed to the corresponding authors.

## Ethics statement

The protocol of this study was approved by Peking University Third Hospital Medical Science Research Ethics Committee (IRB00006761-M2023264). The requirement for written informed consents was waived because all the samples and clinical information used in this study were obtained from a previously established large cohort in our institution. Written informed consents of all individual participants were obtained previously.

## Author contributions

XH: Writing – original draft, Validation, Supervision, Software, Resources, Investigation, Formal analysis, Data curation. XC: Writing – original draft, Validation, Resources, Methodology, Formal analysis. ZZ: Writing – original draft, Resources, Project administration, Data curation, Conceptualization. RW: Writing – original draft, Project administration, Investigation, Formal analysis, Conceptualization. QZ: Writing – original draft, Validation, Methodology, Formal analysis, Data curation. LX: Writing – original draft, Software, Methodology, Formal analysis. HZ: Writing – review & editing, Software, Project administration, Formal analysis. QG: Writing – review & editing, Supervision, Methodology, Formal analysis, Data curation. YL: Writing – review & editing, Visualization, Supervision, Resources, Project administration, Methodology, Investigation, Funding acquisition, Data curation, Conceptualization.

## Glossary

**Table:**

PBMCs	Peripheral blood mononuclear cells
SARS-CoV-2	Severe acute respiratory syndrome coronavirus 2
cDCs	classical dendritic cells
AUC	area under curve
CyTOF	Mass cytometry by time of flight
RT-PCR	Reverse transcription-polymerase chain reaction
UCL	University College London
PUTH	Peking University Third Hospital
FACS	Fluorescence-activated cell sorting
PBS	Phosphate buffered saline
CSB	Cell Staining Buffer
PCA	Principal component analysis
ROC	Receiver operating characteristic
ICU	Intensive Care Unit
FlowSOM	Flow self-organizing map
t-SNE	t-distributed stochastic neighbor embedding analysis
NK cells	Natural killer cells
DCs	Dendritic cells
Tregs	regulatory T cells
CS	Citrate Synthase
GLS	Glutaminase
IDO1	Indoleamine 2,3-dioxygenase 1
KAT1	Kynurenine aminotransferase 1
PFKFB3	6-phosphofructo-2-kinase/fructose-2, 6-biphosphatase 3
Ada	AdaBoost
BP	Back Propagation
GBDT	Gradient Boosting Decision Tree
RF	Random Forest
SVM	Support Vector Machine
WHO	World Health Organization
CRP	C-reactive protein
LDH	lactate dehydrogenase
C3	Complement component 3
C4	Complement component 4
CH50	50% hemolytic unit of complement
IgG	Immunoglobulin G
IgA	Immunoglobulin A
IgM	Immunoglobulin M
IgE	Immunoglobulin E
IL-1	Interleukin-1
IL-2	Interleukin-2
IL-4	Interleukin-4
IL-5	Interleukin-5
IL-6	Interleukin-6
IL-8	Interleukin-8
IL-9	Interleukin-9
IL-10	Interleukin-10
IL-12	Interleukin-12
IL-13	Interleukin-13
IL-17	Interleukin-17
INF-α	Interferon-α
IFN-γ	Interferon-γ
TNF-α	Tumor Necrosis Factor-α
GCSF	Granulocyte colony stimulating factor
GM-CSF	Granulocyte macrophage colony stimulating factor
VEGF	Vascular Endothelial Growth Factor
MIP1-α	Macrophage Inflammatory Protein-1-α
MCP1	Monocyte Chemotactic Protein-1
Tcm	central memory T cell
EMR	electronic medical records
PCT	Procalcitonin.
