# Supplementary material for: A generalizable and easy-to-use COVID-19 stratification model for the next pandemic via immune-phenotyping and machine learning
Source: Front Immunol. 2024 Mar 27;15:1372539. doi: 10.3389/fimmu.2024.1372539 (PMC11004273; doi:10.3389/fimmu.2024.1372539)
Supplement: Supplementary file 1 [file DataSheet_1.docx]

Supplementary Material

# Supplementary Figures and Tables

## Supplementary Figures


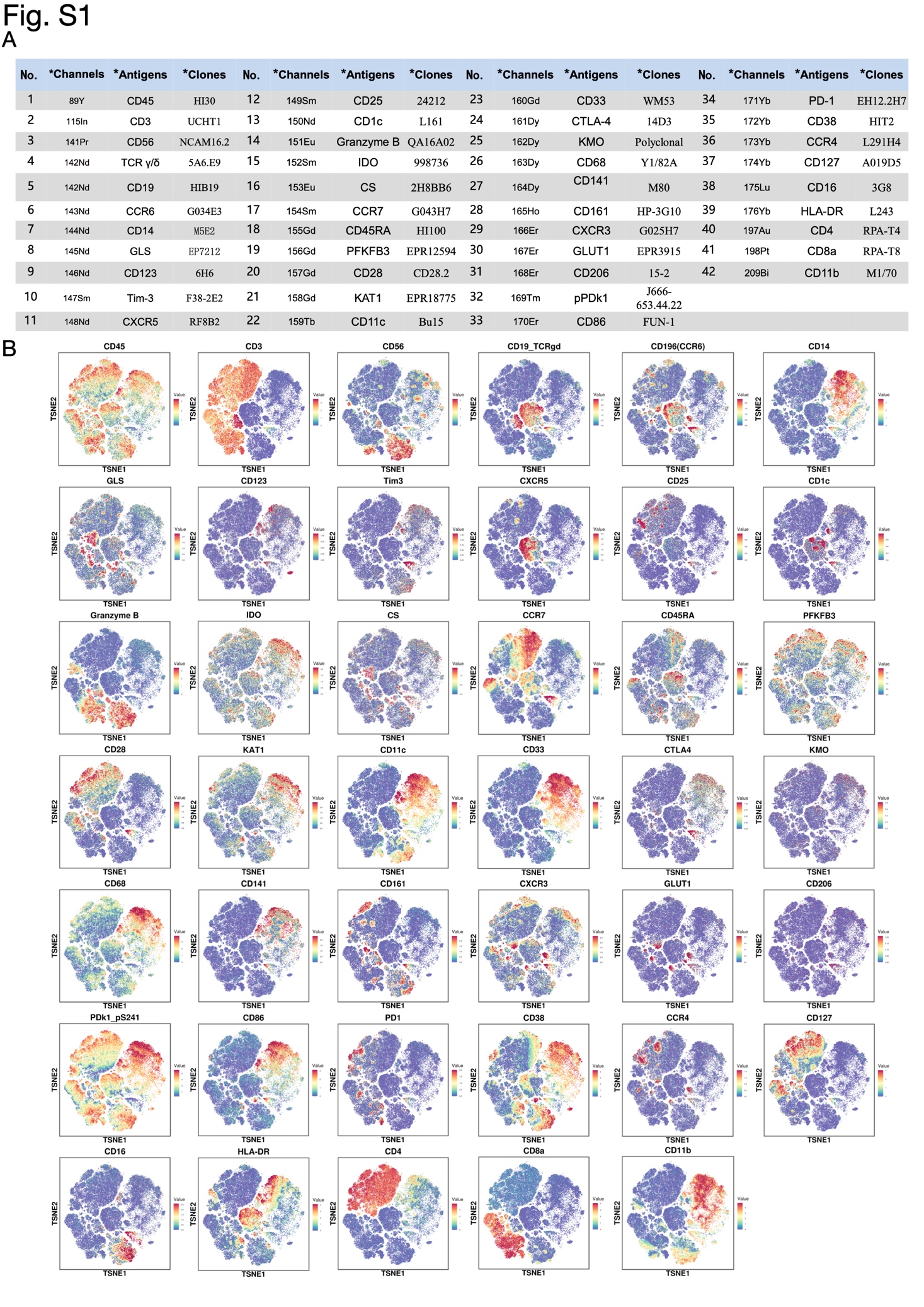


**Figure S1. Detailed information about the antibody panel used for mass cytometry analysis.**

**A.** Detailed information about 42 metal-conjugated antibodies. **B.** Normalized expression of indicated markers in the t-SNE map.


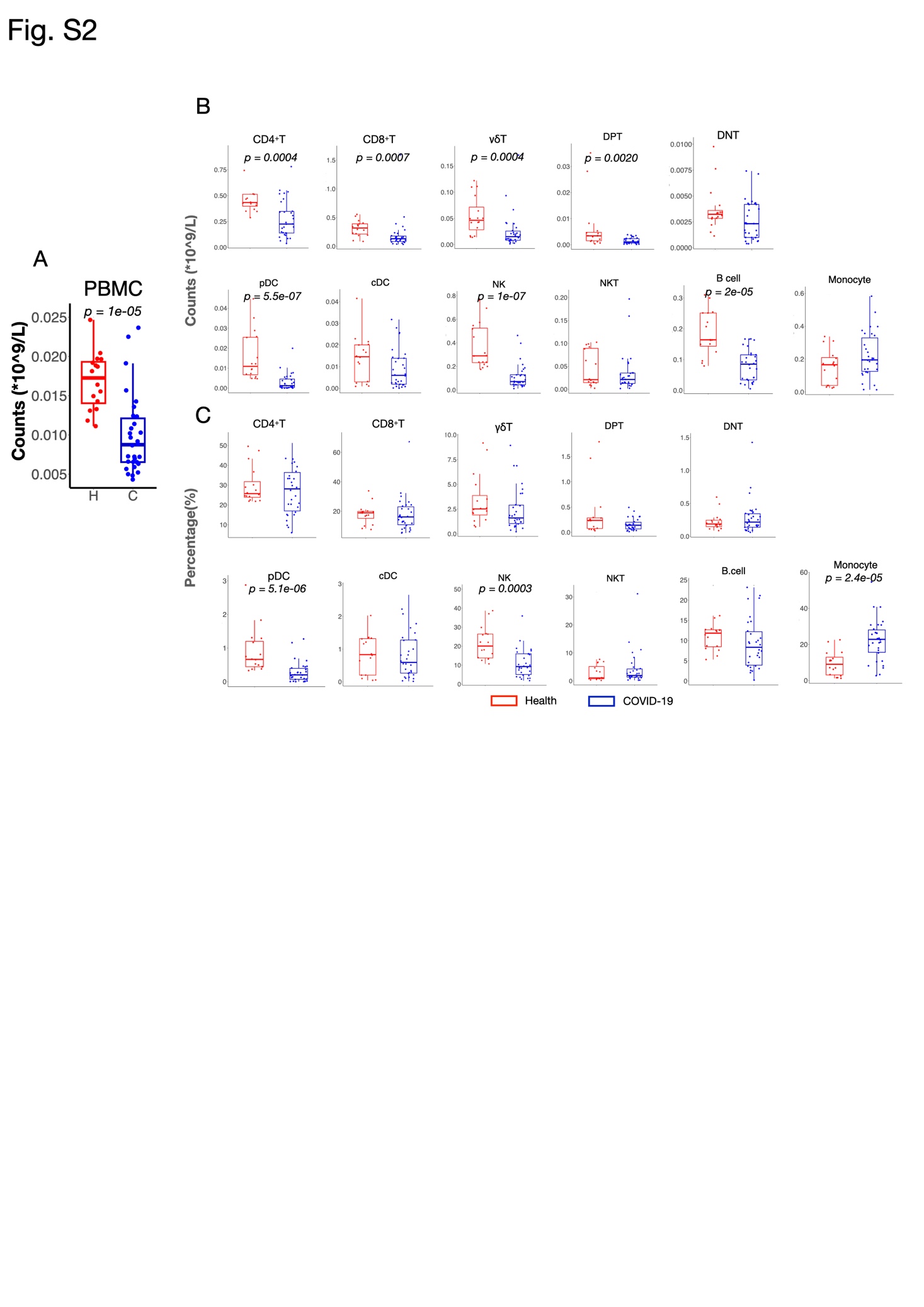


**Figure S2. Differences in counts/percentages of major immune cell populations between patients with COVID-19 and the healthy volunteers**

**A.** Comparison of the total counts of PBMCs at Day 1. **B.** Comparison of the counts of major immune cell populations at Day 1. **C.** Comparison of the percentages of major immune cell populations at Day 1. The center, box and whiskers of the boxplot represent the median, IQR and 1.5 × IQR, respectively. The t-test was used for normally distributed data and the Mann–Whitney U-test was used for non-normally distributed data.


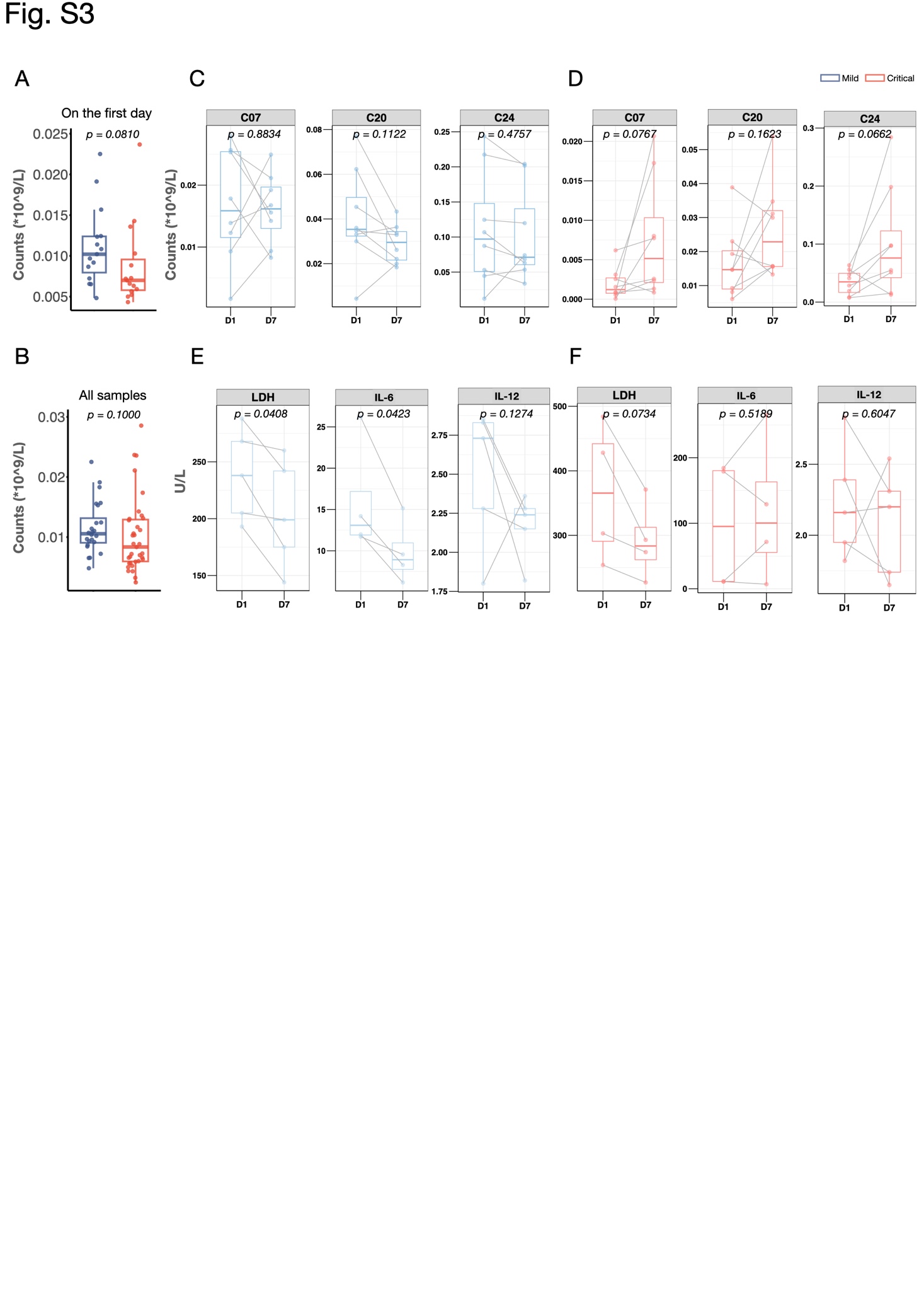


**Figure S3. Dynamic changes in the COVID-19-specific clusters and key cytokines.**

**A, B.** PBMC counts in patients with mild and critical COVID-19 at Day 1 and at all sampling times. The center, box and whiskers of the boxplot represent the median, IQR and 1.5 × IQR, respectively. The t-test was used for normally distributed data and the Mann–Whitney U-test was used for non-normally distributed data. **C, D.** Dynamic changes in COVID-19-specific clusters in patients with mild or critical COVID-19. **E, F.** Dynamic changes in the levels of COVID-19 key clinical laboratory parameters in patients with mild and critical COVID-19. Each line shows the dynamic changes in one patient. Significance was calculated using a paired t-test.


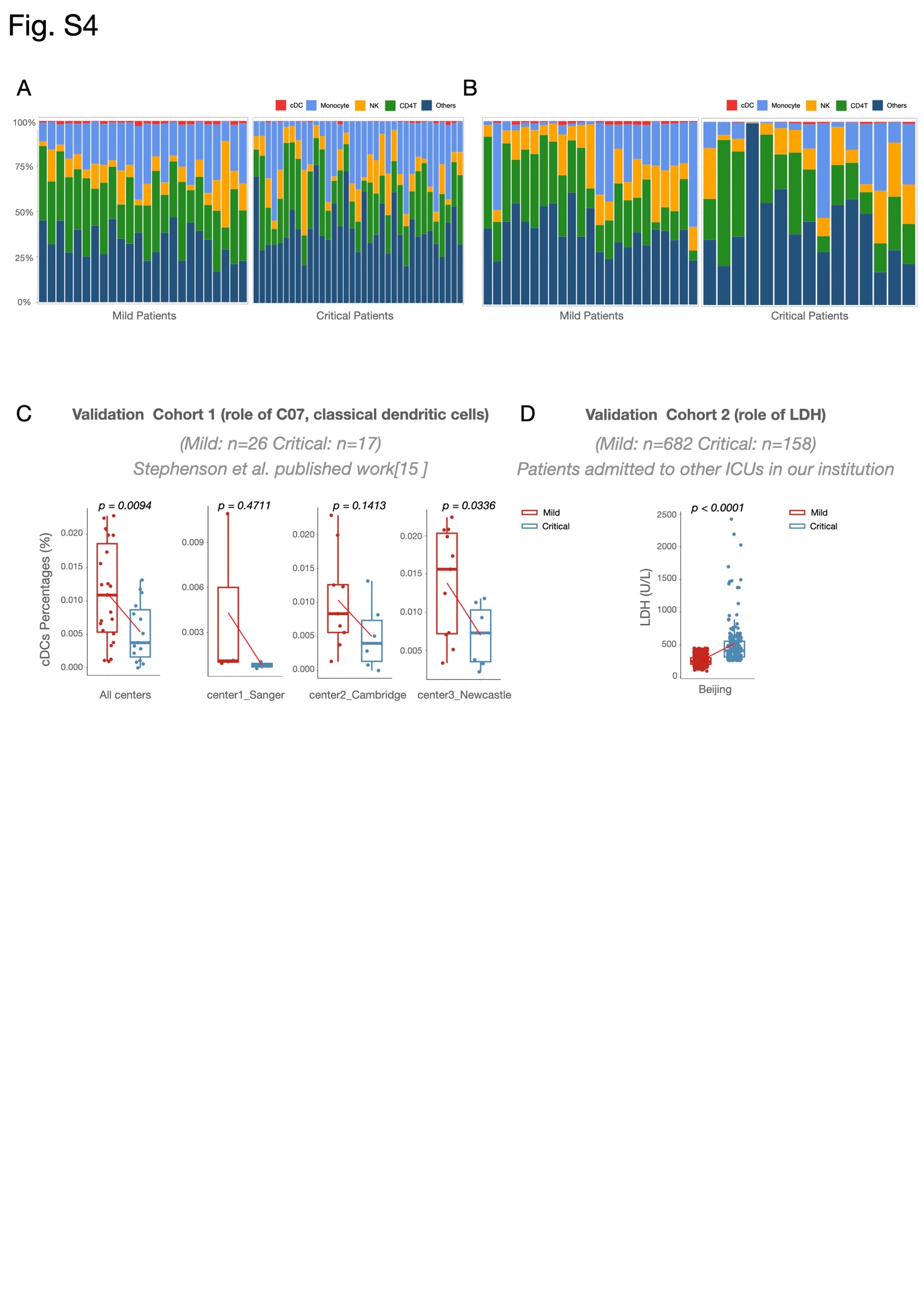


**Figure S4. General information of the validation cohorts.**

**A, B.** Percentage of the immune cell types in patients with COVID-19 in the discovery cohort (**A**) and validation cohort 1 (**B**). **C.** Percentage of cDCs from the three UK centers in the validation cohort 1 of patients with mild and critical COVID-19. **D.** LDH levels of validation cohort 2 in patients with mild and critical COVID-19. The center, box and whiskers of the boxplot represent the median, IQR and 1.5 × IQR, respectively. The t-test was used for normally distributed data and the Mann–Whitney U-test was used for non-normally distributed data.

## Supplementary Tables

| Table S1. Clinical severity classification criteria. | | |
| --- | --- | --- |
| **Severity degree** | **characteristics** | **Definitions** |
| **Mild** | Pneumonia | Patients with clinical signs of pneumonia (fever, cough, dyspnea, fast breathing) but no signs of severe pneumonia, including SpO_2_ ≥ 90% on room air. |
| **Critical** | Severe pneumonia | Patients with clinical signs of pneumonia (fever, cough, dyspnea) plus one of:   - Respiratory rate > 30 breaths/min; - Severe respiratory distress; - Sp0_2_ < 90% on room air.   While the diagnosis can be made on clinical grounds; chest imaging (radiograph, CT scan, ultrasound) may assist in diagnosis and identify or exclude pulmonary complications |
|  | Acute Respiratory Distress Syndrome  (ARDS) | Onset: within 1 week of a known clinical insult (i.e. pneumonia) or new or worsening respiratory symptoms.  Chest imaging: (radiograph, CT scan, or lung ultrasound): bilateral opacities, not fully explained by volume overload, lobar or lung collapse, or nodules. Origin of pulmonary infiltrates: respiratory failure not fully explained by cardiac failure or fluid overload. Need objective assessment (e.g. echocardiography) to exclude hydrostatic cause of infiltrates/oedema if no risk factor present. Oxygenation impairment:   - Mild ARDS: 200 mmHg < PaO_2_/FiO_2_ ≤ 300 mmHg (with PEEP or CPAP ≥ 5 cmH_2_O). - Moderate ARDS: 100 mmHg < PaO_2_/FiO_2_ ≤ 200 mmHg (with PEEP ≥ 5 cmH_2_O). - Severe ARDS: PaO_2_/FiO_2_ ≤ 100 mmHg (with PEEP ≥ 5 cmH_2_O). |
|  | Sepsis | Acute life-threatening organ dysfunction caused by a dysregulated host response to suspected or proven infection. Signs of organ dysfunction include: altered mental status (delirium), difficult or fast breathing, low oxygen saturation, reduced urine output, fast heart rate, weak pulse, cold extremities or low blood pressure, skin mottling, laboratory evidence of coagulopathy, thrombocytopenia, acidosis, high lactate, or hyperbilirubinemia. |
|  | Septic shock | Persistent hypotension despite volume resuscitation, requiring vasopressors to maintain MAP ≥ 65 mmHg and serum lactate level > 2 mmol/L. |
|  | Acute thrombosis | Acute venous thromboembolism (i.e. pulmonary embolism), acute coronary syndrome, acute stroke. |

Abbreviations: SpO_2_, oxygen saturation; CT, computed tomography; PaO_2_, arterial partial pressure of oxygen; FiO_2_, fraction of inspired oxygen; CPAP, continuous positive airway pressure; PEEP, positive end-expiratory pressure; MAP, mean arterial pressure.

| Table S2. Detailed information on the antibody panel used for mass cytometry analysis | | | | | | |
| --- | --- | --- | --- | --- | --- | --- |
| **List** | **Label** | **Marker** | **Clone** | **Dilution** | **Source** | **Identifier** |
| 1 | 89Y | CD45 | HI30 | 200 | Biolegend | Cat#304002 |
| 2 | 115In | CD3 | UCHT1 | 100 | Bioxcell | Cat#BE0231 |
| 3 | 141Pr | CD56 | NCAM16.2 | 400 | BD | Cat#559043 |
| 4 | 142Nd | TCR γ/δ | 5A6.E9 | 200 | PLT | Cat#100P001A |
| 5 | 142Nd | CD19 | HIB19 | 400 | Biolegend | Cat#302268 |
| 6 | 143Nd | CD196(CCR6) | G034E3 | 200 | Biolegend | Cat#353402 |
| 7 | 144Nd | CD14 | M5E2 | 100 | Biolegend | Cat#301862 |
| 8 | 145Nd | GLS(Glutaminase) | EP7212 | 100 | Abcam | Cat#ab214802 |
| 9 | 146Nd | CD123(IL-3Rα) | 6H6 | 200 | Biolegend | Cat#306002 |
| 10 | 147Sm | CD366(Tim-3) | F38-2E2 | 50 | Biolegend | Cat#345010 |
| 11 | 148Nd | CD185(CXCR5) | RF8B2 | 400 | BD | Cat#552032 |
| 12 | 149Sm | CD25(IL-2Rα) | 24212 | 200 | RD | Cat#MAB1020 |
| 13 | 150Nd | CD1c | L161 | 200 | Biolegend | Cat#331502 |
| 14 | 151Eu | Granzyme B Recombinant | QA16A02 | 200 | Biolegend | Cat#372202 |
| 15 | 152Sm | IDO(Indoleamine 2,3-dioxygenase) | 998736 | 100 | RD | Cat#MAB60302-100 |
| 16 | 153Eu | CS(Citrate synthetase) | EPR8067 | 100 | Abcam | Cat#ab233838 |
| 17 | 154Sm | CD197(CCR7) | G043H7 | 50 | Biolegend | Cat#353256 |
| 18 | 155Gd | CD45RA | HI100 | 800 | Biolegend | Cat#304102 |
| 19 | 156Gd | PFKFB3 | EPR12594 | 100 | Abcam | Cat#ab218121 |
| 20 | 157Gd | CD28 | CD28.2 | 100 | Biolegend | Cat#302934 |
| 21 | 158Gd | KAT1(HAT1) | EPR18775 | 100 | Abcam | Cat#ab251185 |
| 22 | 159Tb | CD11c | Bu15 | 200 | Biolegend | Cat#337202 |
| 23 | 160Gd | CD33 | WM53 | 800 | Biolegend | Cat#303419 |
| 24 | 161Dy | CD152(CTLA-4) | 14D3 | 100 | eB | Cat#14-1529-82 |
| 25 | 162Dy | KMO | Polyclonal | 200 | Thermo | Cat#PA535353 |
| 26 | 163Dy | CD68 | Y1/82A | 100 | Biolegend | Cat#333802 |
| 27 | 164Dy | CD141(Thrombomodulin) | M80 | 200 | Biolegend | Cat#344102 |
| 28 | 165Ho | CD161 | HP-3G10 | 100 | Biolegend | Cat#339902 |
| 29 | 166Er | CD183(CXCR3) | G025H7 | 200 | Biolegend | Cat#353750 |
| 30 | 167Er | GLUT1 | EPR3915 | 200 | RD | Cat#MAB1418 |
| 31 | 168Er | CD206(MMR) | 15-2 | 200 | Biolegend | Cat#321150 |
| 32 | 169Tm | PDk1(Phospho-Ser241) | J66-653.44.22 | 200 | Biorbyt | Cat#orb105930 |
| 33 | 170Er | CD86 | FUN-1 | 200 | BD | Cat#555655 |
| 34 | 171Yb | CD279(PD-1) | EH12.2H7 | 200 | Biolegend | Cat#329926 |
| 35 | 172Yb | CD38 | HIT2 | 100 | Biolegend | Cat#303502 |
| 36 | 173Yb | CD194(CCR4） | L291H4 | 400 | Biolegend | Cat#359402 |
| 37 | 174Yb | CD127(IL-7Rα) | A019D5 | 100 | Biolegend | Cat#351302 |
| 38 | 175Lu | CD16 | 3G8 | 100 | Biolegend | Cat#302057 |
| 39 | 176Yb | HLA-DR | L243 | 200 | Biolegend | Cat#307648 |
| 40 | 197Au | CD4 | RPA-T4 | 400 | Biolegend | Cat#300570 |
| 41 | 198Pt | CD8a | RPA-T8 | 200 | Biolegend | Cat#301074 |
| 42 | 209Bi | CD11b | M1/70 | 800 | Biolegend | Cat#101202 |

| Table S3. Advantages of Ada, BP, GBDT, RF, SVM | | |
| --- | --- | --- |
| **Types of Machine Learning** | **Machine Learning algorithms** | **Advantages** |
| **Supervised Learning** | **AdaBoost**  **(Ada)** | - Powerful classifier - Capable of handling high-dimensional and complex problems - Resistant to overfitting |
|  | **Back Propagation**  **(BP)** | - Suitable for complex non-linear relationships - Automatic feature learning - Strong capability in image - Speech recognition |
|  | **Gradient Boosting Decision Tree**  **(GBDT)** | - High accuracy - Capable of handling large-scale data - Resistant to overfitting - Strong capability in handling non-linear problems |
|  | **Random Forest**  **(RF)** | - High accuracy - Capable of processing large-scale data - Resistant to overfitting - Strong capability in dealing with high feature dimensions |
|  | **Support Vector Machine**  **(SVM)** | - Capable of handling non-linear data - Strong capability in high-dimensional space - Effective on smaller datasets |

| Table S4. Strategies for major immune cell populations identification | |
| --- | --- |
| **Cell populations** | **Lineage markers** |
| Monocytes | CD11b^+^CD14^+^ |
| cDCs | CD11b^+^CD11c^+^CD14^-^ |
| pDCs | CD11b^+^CD11c^+^CD123^+^ |
| NK | CD3^-^CD56^+^ |
| CD4^+^T | CD3^+^CD4^+^CD8^-^ |
| CD8^+^T | CD3^+^CD8^+^CD4^-^ |
| DNT | CD3^+^CD4^-^CD8^-^ |
| DPT | CD3^+^CD4^+^CD8^+^ |
| γδT | CD3^+^TCRγδ^+^ |
| NKT | CD3^+^CD56^+^ |
| B | CD19^+^ |

| Table S5. Components and individual values of the lung injury score | | |
| --- | --- | --- |
|  | **value** | |
| 1. Chest roentgenogram score |  |  |
| No alveolar consolidation |  | 0 |
| Alveolar consolidation confined to 1 quadrant |  | 1 |
| Alveolar consolidation confined to 2 quadrants |  | 2 |
| Alveolar consolidation confined to 3 quadrants |  | 3 |
| Alveolar consolidation in all 4 quadrants |  | 4 |
| 2. Hypoxemia score |  |  |
| PaO_2_/FiO_2_ | ≥300 | 0 |
| PaO_2_/FiO_2_ | 225-299 | 1 |
| PaO_2_/FiO_2_ | 175-224 | 2 |
| PaO_2_/FiO_2_ | 100-174 | 3 |
| PaO_2_/FiO_2_ | ＜100 | 4 |
| 3. PEEP score (when ventilated) |  |  |
| PEEP | ≥5 cm H_2_O | 0 |
| PEEP | 6-8 cm H_2_O | 1 |
| PEEP | 9-11 cm H_2_O | 2 |
| PEEP | 12-14 cm H_2_O | 3 |
| PEEP | ≥15 cm H_2_O | 4 |
| 4. Respiratory system compliance score (when ventilated) |  |  |
| Compliance | ≥80 ml/cm H_2_O | 0 |
| Compliance | 60-79 ml/cm H_2_O | 1 |
| Compliance | 40-59 ml/cm H_2_O | 2 |
| Compliance | 20-39 ml/cm H_2_O | 3 |
| Compliance | ≤19 ml/cm H_2_O | 4 |
| The final value is obtained by dividing the aggregate sum by the number of components used | | |
|  | score | |
| No lung injury | 0 | |
| Mild-to-moderate lung injury | 0.1-2.5 | |
| Severe lung injury (ARDS) | ＞2.5 | |

Abbreviations: PaO_2_/FiO_2_: arterial oxygen tension to inspired oxygen concentration ration; PEEP: positive pressure; ARDS: acute respiratory distress syndrome.
